# Submicron Dispersions of Phytosterols Reverse Liver Steatosis with Higher Efficacy than Phytosterol Esters in a Diet Induced-Fatty Liver Murine Model

**DOI:** 10.3390/ijms26020564

**Published:** 2025-01-10

**Authors:** Raimundo Gillet, Tomás G. Cerda-Drago, María C. Brañes, Rodrigo Valenzuela

**Affiliations:** 1 Naturalis Research Consortium, Santiago 8700548, Chile; raimundo.gillet@nutrartis.com (R.G.); tomasgcd@gmail.com (T.G.C.-D.); cbranes@harting.cl (M.C.B.); 2 Department of Nutrition, Faculty of Medicine, University of Chile, Santiago 8380000, Chile

**Keywords:** phytosterols, MAFLD, dyslipidemia, liver steatosis

## Abstract

Consumption of phytosterols is a nutritional strategy employed to reduce cholesterol absorption, but recent research shows that their biological activity might go beyond cholesterol reduction for the treatment of metabolic dysfunction-associated fatty liver disease (MAFLD), and novel phytosterol formulations, such as submicron dispersions, could improve these effects. We explored the therapeutic activity of phytosterols, either formulated as submicron dispersions of phytosterols (SDPs) or conventional phytosterol esters (PEs), in a mouse model of MAFLD. MAFLD was induced in mice by atherogenic diet (AD) feeding. The reversion of distorted serum and liver parameter values after a period of AD feeding was investigated after supplementation of the AD with SDPs, PEs, or a placebo (PT). Additionally, the metabolic parameters of fatty acid synthesis, fatty acid oxidation, and inflammation were studied to understand the mechanism of action of phytosterols. AD supplementation with SDPs was shown to reduce liver fat, along with showing a significant improvement in liver triglycerides (TGs), free fatty acids (FFAs), and liver cholesterol levels. These results were reinforced by the analyses of the liver steatosis scores, and liver histologies, where SDP intervention showed a consistent improvement. Treatment with PEs showed slighter effects in the same analyses, and no effects were observed with the PT treatment. Additionally, SDP intervention reversed, with a higher efficacy than PEs, the effect of AD on the serum levels of TGs, total- and LDL-cholesterol levels, and glucose levels. And, exceptionally, while SDP improved HDL-cholesterol serum levels, PEs did not show any effect on this parameter. We provide evidence for the therapeutical activity of phytosterols in MAFLD beyond the regulation of cholesterol levels, which is increased when the phytosterols are formulated as submicron dispersions compared to ester formulations.

## 1. Introduction

Metabolic dysfunction-associated fatty liver disease (MAFLD), also known as nonalcoholic fatty liver disease (NAFLD), is a condition characterized by several changes in the normal liver functions related to steatosis, inflammation, necrosis, and various grades of fibrosis [1]. The term MAFLD refers to hepatic steatosis in addition to overweight/obesity, or the presence of type 2 diabetes mellitus, or evidence of metabolic dysregulation [2]. It involves liver abnormalities from steatosis, known as an accumulation of more than 5% of ectopic fat in the liver, to steatohepatitis, an inflammatory subtype of MAFLD that involves excessive fat accumulation in the liver, which leads to an increasing inflammatory state that may progress to fibrosis and cirrhosis, generating livery dysfunction [3]. Liver steatosis progression to steatohepatitis is caused by excessive triglycerides (TGs) storage and free fatty acids in the hepatocyte, giving rise to excessive lipid peroxidation as a result of the production of reactive oxygen species [4]. Almost 25% of the world’s population is affected by MAFLD [5] and it is one of the causes of chronic liver disease, causing most of the recurrences worldwide [6].

The origin and the development of MAFLD are complex. Part of the disease originates from fat accumulation in this organ, but it affects other tissues and organs through a complex network of biochemical mechanisms. One of them is insulin resistance, which can lead to increased de novo lipogenesis and the impaired inhibition of adipose tissue lipolysis, resulting in an increased flux of fatty acids to the liver [7]. Adipose tissue dysfunction can lead to the altered secretion of inflammatory cytokines [8]. Furthermore, fat accumulation in the liver in the form of TGs and other lipid metabolites such as free fatty acids and cholesterol leads to increased lipotoxicity, resulting in organelle damage mostly represented by endoplasmic reticulum stress and mitochondrial dysfunction [9]. Another factor is the development of an altered microbiota, which has been observed to affect energy balance and to produce the microbial metabolites that are involved in the development of MAFLD [10]. Thus, the aforementioned factors that form a complex network in the development of MAFLD have been studied as potential therapeutic targets for treating the disease.

MAFLD is hypothesized to emerge from both genetical predisposition, and lifestyle-related factors such as bad dietary habits, sedentarism, and toxicity produced by environmental chemicals [11]. Starting from this hypothesis, therapy for MAFLD should be oriented to lifestyle changes [12], as well as to treatments for preventing or reversing hepatocellular damage through the inhibition of oxidative stress, lipid peroxidation, and inflammation [13]. The available pharmacological treatments for MAFLD are aimed at the correction of the associated metabolic disorders (statins, antihypertensive agents, antidiabetic drugs, etc.) [14]. However, these treatments are limited due to the complex network of factors that drive MAFLD pathogenesis [15]. The only approved drug for nonalcoholic steatohepatitis (NASH) is not preventive and is only applied in the late stages of the disease [16].

In addition to drugs, natural products with metabolic effects have been praised as candidates to reduce the effects of MAFLD. In particular, phytosterols and phytostanols have aroused interest for their cholesterol-lowering properties, which come without side effects. Together with the potential benefits related to the reversal of the pathogenesis of MAFLD, these methods could provide a relevant strategy for MAFLD prevention or reversion [17]. From a mechanistic point of view, phytosterols can interact directly or indirectly with the nuclear receptors that are involved in lipid metabolism, carbohydrate metabolism, and inflammation [18]. Experiments in animals have demonstrated the possible MAFLD pathogenesis-related effects of phytosterols such as the reduction of plasma and the liver levels of TGs, cholesterol, and free fatty acids [19,20,21], the modulation of oxidative stress and endoplasmic reticulum stress markers [21,22], a reduction in mitochondrial damage [23], an interaction with the mediators of fatty acid and cholesterol synthesis [21,23], a direct effect on macrophage inflammation as well as an indirect effect through the modulation of pro-inflammatory markers [20,21,22], and beneficial changes in microbiota for the treatment of MAFLD [24,25]. Improvements have been clinically demonstrated as well in several markers such as lipid profiles, liver enzymes, fasting glucose levels, and inflammatory markers [26,27,28,29].

Nevertheless, clinical studies using only phytosterols as free [26,29] or esterified [27,28] forms have not been able to demonstrate a clear MAFLD reduction in humans. Contrary to this trend, recent pilot-scale evidence shows that phytosterols, when formulated as submicron water-dispersible particles, were able to produce a median relative change of 19% in liver steatosis for a group of 26 patients with NASH [30]. In a similar line, in previous works, submicron dispersions of phytosterols (SDPs) have been reported to present additional effects to the known LDL-cholesterol reducing effect of phytosterol esters (PEs), such as TGs reduction and HDL-cholesterol improvement in mildly-hypercholesterolemic patients [31], and improved waist circumferences and bowel movements in metabolic-syndrome patients [32].

In the present study, in vivo experimental models of animals with MAFLD were treated with phytosterols formulated as submicron dispersions or as esters, to assess the potential different functionalities according to each formulation, and the possible mechanisms of action of phytosterols and their therapeutic effects in relation to lipid metabolism, glucose metabolism, and inflammation, were discussed.

## 2. Results

### 2.1. Macro and Micro (or Metabolic) Effects of AD

MAFLD development was confirmed after 4 weeks through a liver biopsy in one animal of each group. An evaluation of the state of the animals after consuming AD (Table 1) showed no differences in the average final animal weights among all of the treatments. Throughout the whole period, the animals presented an average weight gain of 4 g. Similarly, no changes were observed between the average initial and final diet intakes. Following the same trend as the aforementioned parameters, the liver weight and visceral adipose tissue weight did not present any differences among the different treatments.

**Figure 1:**
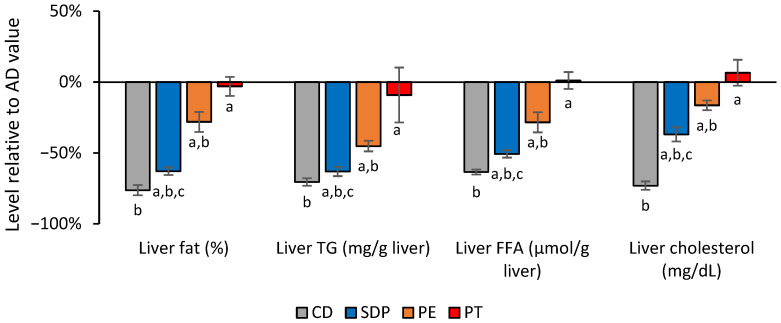
Effect of different treatments in the reduction of lipid liver parameters normalized to the values obtained in the AD group. Values for liver fat, triglycerides (TGs), free fatty acids (FFAs), and cholesterol, obtained in the experimental groups of the animals treated with CD, AD supplemented with SDPs, phytosterol esters (PEs), PT, are normalized with respect to the levels obtained in the AD experimental group (AD = 0%). Results correspond to the average ± SD of three independent experiments that were carried out using *n* = 5 in each experimental group. In this figure, a: *p* < 0.05 versus the CD group; b: *p* < 0.05 versus the PT group; and c: *p* < 0.05 when comparing SDP group to PE group.

**Figure 2:**
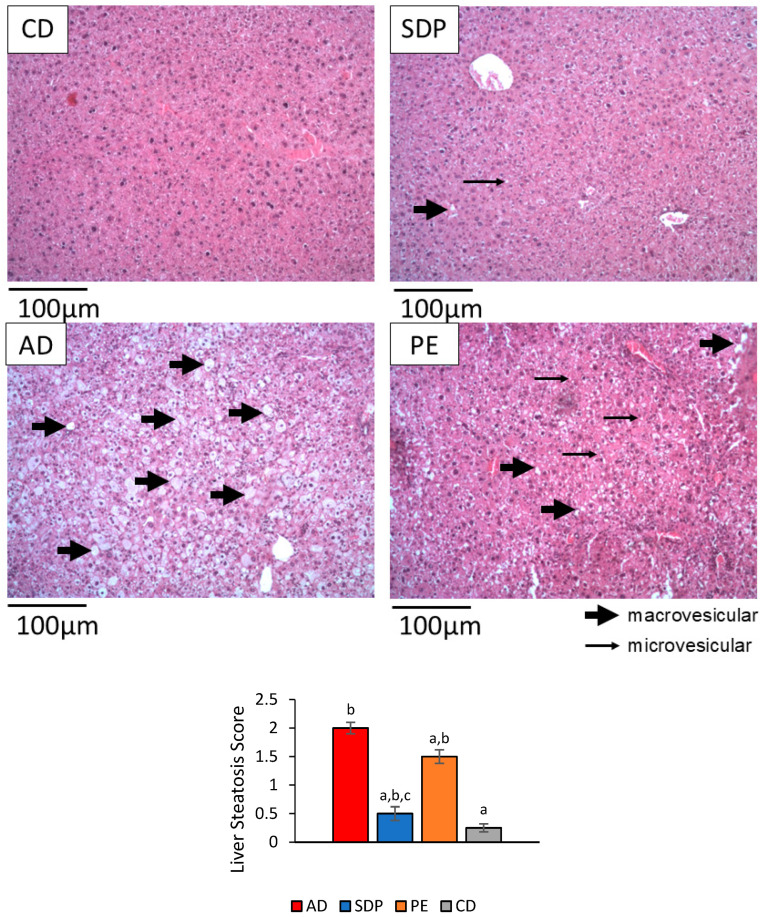
Effect of phytosterol treatments on liver steatosis. Representative images of liver histologies 10× with hematoxylin and eosin staining for the experimental groups of animals treated with atherogenic diet (AD), AD supplemented with submicron dispersion of phytosterols (SDPs), or phytosterol esters (PEs), and control diet (CD). In the image, arrows indicate fat droplets: thick arrow = macrovesicular; thin arrow = microvesicular; n = 5 in each experimental group. Fat globules are depicted with arrows. The graph below shows the quantitative liver steatosis score analysis, where one liver section was considered per mouse. Each bar corresponds to the average ± SD of three independent experiments that were carried out using n = 5 in each experimental group. Liver steatosis scores were evaluated according to Brunt et al., 1999, as a percent of hepatocytes showing macrovesicular steatosis (0 is none, 1 is up to 33%, 2 is 33–66%, and 3 is >66%) [33]. In the figure, a: *p* < 0.05 versus the AD group; b: *p* < 0.05 versus the CD group; and c: *p* < 0.05 when comparing SDP group to PE group.

Despite no effect on liver weight being observed, the administration of AD to the mice induced a significant increase in liver steatosis and an increase in TGs, free fatty acids (FFAs), and total cholesterol liver content, as compared to CD treatment (Figure 1 PT and CD, Figure 2 CD and AD). The administration of AD to the mice also induced a significant increase in the serum parameters related to lipid and carbohydrate metabolism, namely, serum TGs, total-, LDL-, and HDL-cholesterol levels, and glucose, as compared to CD treatment.

### 2.2. Improved Efficacy of SDP Compared to PE in the Treatment of AD-Induced Liver Steatosis and in the Reversion of Altered Liver Lipid Metabolism Parameters In Vivo

The analysis of the effect of each treatment confirmed that placebo (PT) did not modify AD-induced increases in liver fat levels. Nevertheless, a notorious and statistically significant improvement was observed with SDPs compared to an AD (Figure 1 SDP and PT), although this improvement did not reach the levels observed with CD (Figure 1 CD).

After confirming that SDPs improved the deteriorated lipid state of the liver generated by AD consumption even if the animals were still fed an AD, we compared the head-to-head efficacy of PEs and SDPs. Although SDPs and PEs were both effective treatments, the effect of SDPs was significantly better than that of PEs (Figure 1 SDP and PE).

Moreover, the effect of an AD, a CD, SDPs, and PEs on hepatic steatosis was evaluated by HE staining of liver tissue sections. The representative results in Figure 2 show that the mice fed an AD were characterized by significant steatosis with enlarged liver cells filled with macro and micro white lipid droplets in the cytoplasm, as well as a pushing of the nucleus to the side, as compared to the mice fed a CD (Figure 2 AD and CD). While in the liver sections of the mice supplemented with PEs, less and smaller sized lipid droplets were observed. and tissue sections from the SDP-treated animals showed almost no lipid content and closely resembled the control tissue sections (Figure 2 PE, SDP and CD). The liver steatosis score analysis revealed that AD treatment produced an increase to a value of ca. 2 in this parameter, as compared to the ca. 0.25 points observed with CD treatment. SDP supplementation for the AD-consuming mice was observed to significantly reduce liver steatosis scores to ca. 0.75, as compared to a smaller reduction of the score to ca. 1.5 points in the case of PE supplementation for the AD-consuming mice (Figure 2).

**Figure 3:**
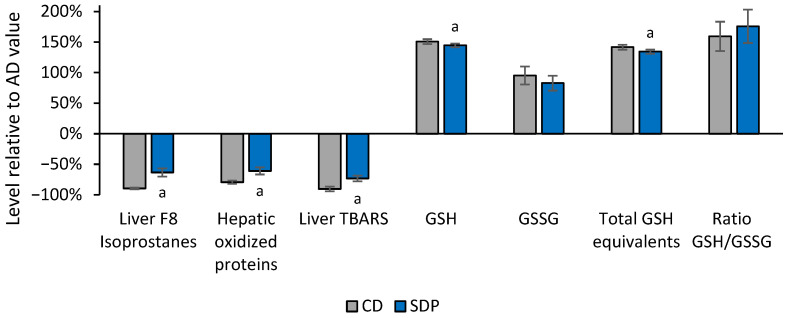
Effect of different treatments in the reduction of liver oxidative stress parameters normalized to the values obtained in the AD group. Values in Liver F8 isoprostanes, hepatic oxidized proteins, liver thiobarbituric acid reactive substances (TBARSs), reduced glutathione (GSH), glutathione disulfide (GSSG), total GSH equivalents, and the ratio of GSH/GSSG, obtained in the experimental groups of animals treated with CD, or AD supplemented with SDPs, are normalized with respect to levels obtained in the AD experimental group (AD = 0%). Each bar represents the average ± SD of three independent experiments that were carried out using *n* = 5 in each experimental group. In the figure, a: *p* < 0.05 versus the CD group.

To explore the mechanisms involved in liver fat reduction induced by SDPs, oxidative stress parameters, namely liver F8 isoprostanes, hepatic oxidized proteins, liver thiobarbituric acid reactive substances (TBARS), reduced glutathione (GSH), glutathione disulfide (GSSG), total GSH equivalents, and the ratio of GSH/GSSG, were also assessed. AD consumption induced an increase in these parameters, which was not observed with CD consumption, and was reversed with SDP supplementation to an AD, almost to the level of the CD (Figure 3 CD and SDP).

### 2.3. Improved Efficacy of SDP Compared to PT and PE on Serum Metabolic Parameters In Vivo

Once it was determined that phytosterols formulated as SDPs had an improved therapeutical effect on MAFLD compared to PEs, we addressed the comparison of the impact of both formulations on serum lipids, and glucose.

**Figure 4:**
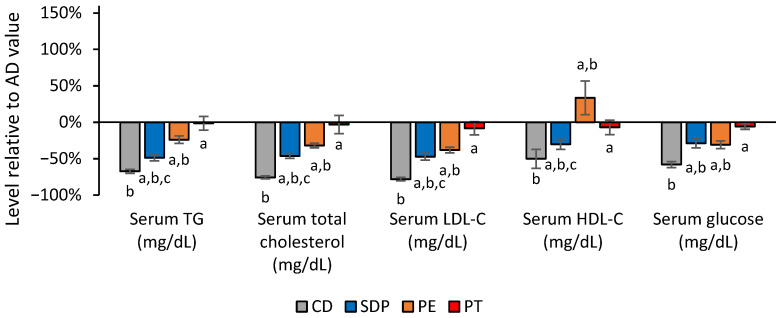
Effect of different treatments on serum lipid and carbohydrate metabolism parameters normalized to the values obtained in the AD group. Values in serum parameters related to lipid and carbohydrate metabolism obtained in the experimental groups of animals treated with CD, AD supplemented with SDPs, or PEs, or PT, are normalized with respect to the levels obtained in the AD experimental group. Each bar represents the average ± SD of three independent experiments that were carried out using *n* = 5 in each experimental group. In the figure, a: *p* < 0.05 versus the CD group; b: *p* < 0.05 versus the PT group; and c: *p* < 0.05 when comparing SDP group to PE group.

As detected in the liver parameters, SDP treatment produced significant improvements in the levels of the serum metabolic parameters which were altered after AD treatment. These included serum lipids and glucose (Figure 4 SDP). Meanwhile, PEs resulted in a significantly lower efficacy in the serum lipids, and notably, PE did not produce any significant change in HDL-cholesterol levels while SDP was active (Figure 4 SDPs and PEs). Both formulations showed equivalent effects on glucose levels (Figure 4 PE).

**Figure 5:**
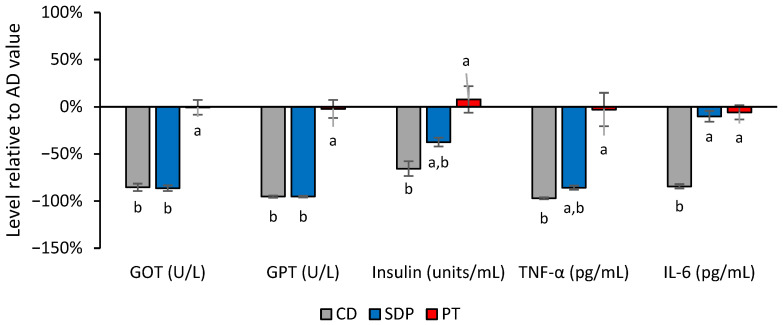
Effect of different treatments on serum transaminases, inflammation parameters, and insulin, normalized to the values obtained in the AD group. Serum transaminases, inflammation parameters, and insulin values obtained in the experimental groups of animals treated with CD, AD supplemented with SDPs, or PT, normalized with respect to the levels obtained in the AD experimental group. Each bar represents the average ± SD of three independent experiments that were carried out using *n* = 5 in each experimental group. In the figure, a: *p* < 0.05 versus the CD group and b: *p* < 0.05 versus the PT group.

Considering the positive results obtained in the SDP group, its biological effect was studied with deeper insight by analyzing insulin, aspartate aminotransferase (GOT), alanine aminotransferase (GPT), TNF-α, and IL-6 serum levels and comparing with the PT group (Figure 5 SDP). AD consumption induced an increase in these parameters, which was not observed with CD consumption, and was reversed with SDP supplementation to an AD, almost to the level of the CD group (Figure 5 PT, CD, and SDP).

### 2.4. Efficacy of Submicron Dispersible Phytosterols Is Reflected in the Regulation of Metabolic and Inflammatory Responses

**Figure 6:**
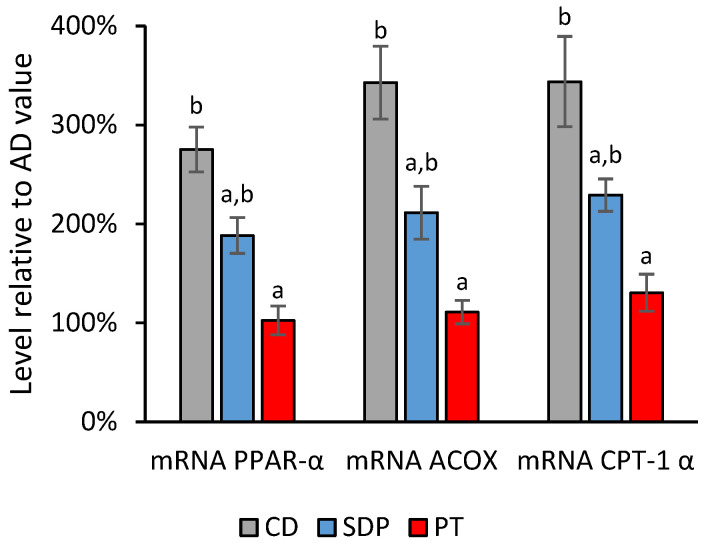
Effect of different treatments on transcript levels of fatty acid oxidation markers normalized to the values obtained in the AD group. Transcript levels of genes linked to the oxidation of fatty acids and obtaining energy (PPAR-α, CPT-1, ACOX) observed in the experimental groups of animals treated with AD supplemented with SDPs, or PT, and CD, normalized with respect to the levels obtained in the AD experimental group. Each bar represents the average ± SD of three experimental replicas that were carried out using *n* = 5 in each experimental group. In the figure, a: *p* < 0.05 versus the AD group and b: *p* < 0.05 versus the CD group.

**Figure 7:**
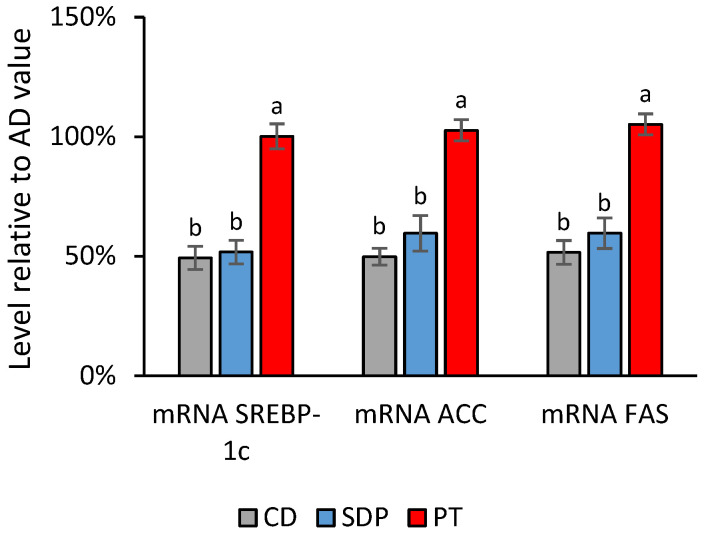
Effect of different treatments on transcript levels of fatty acids synthesis markers normalized to the values obtained in the AD group. Transcript levels of genes linked to the synthesis of fatty acids (SREBP-1c, ACC, FAS) observed in the experimental groups of animals treated with AD supplemented with SDPs, or PT, and CD, normalized with respect to the levels obtained in the AD experimental group. Each bar represents the average ± SD of three experimental replicas that were carried out using *n* = 5 in each experimental group. In the figure, a: *p* < 0.05 versus the AD group and b: *p* < 0.05 versus the CD group.

**Figure 8:**
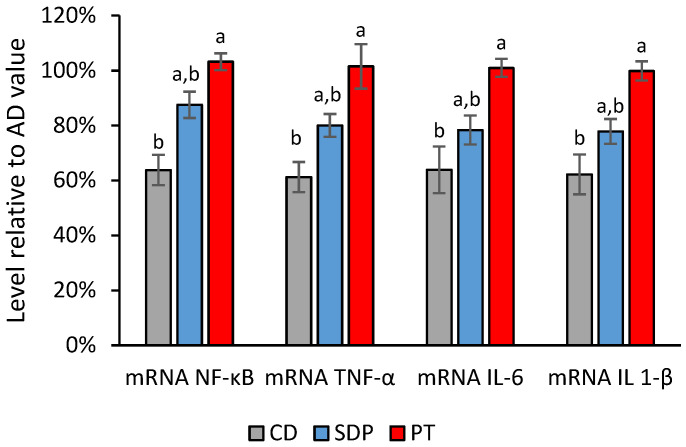
Effect of different treatments on transcript levels of pro-inflammatory markers normalized to the values obtained in the AD group. Transcript levels of genes linked to inflammatory responses (NF-κB, TNF-α, IL-6, IL-1β) observed in the experimental groups of animals treated with AD supplemented with SDPs, or PT, and CD, normalized with respect to the levels obtained in the AD experimental group. Each bar represents the average ± SD of three experimental replicas that were carried out using *n* = 5 in each experimental group. In the figure, a: *p* < 0.05 versus the AD group and b: *p* < 0.05 versus the CD group.

In search of the potential mechanistic targets of phytosterols, the transcript levels of gene markers and the enzymatic activities of lipid metabolisms, and inflammation responses were analyzed in the livers after each treatment. This analysis included the following groups of genes: (i) genes linked to the oxidation of fatty acids and energy obtention (peroxisome proliferator-activated receptor alpha, PPAR-α; carnitine-palmitoyl transferase-1, CPT-1; and acyl-CoA oxidase, ACOX); (ii) genes linked to fatty acid synthesis (sterol regulatory element-binding protein-1c, SREBP-1c; acetylCoA carboxylase, ACC; and fatty acid synthase, FAS); and (iii) genes linked to the inflammatory responses (nuclear factor kappa B, NF-κB; tumor necrosis factor alpha, TNF-α; interleukin 6, IL-6; and interleukin 1 beta, IL-1β). AD administration to the mice induced a decrease in the transcript levels of all the gene markers related to fatty acid oxidation, and an increase in the transcript levels of all the gene markers for fatty acid synthesis and inflammation, as compared to CD administration. Conversely, SDPs significantly reversed the effect of an AD on all of these parameters, and tended to normalize them to the levels of the CD group, while PT did not produce any reversion akin to the effect of an AD (Figure 6, Figure 7 and Figure 8).

**Figure 9:**
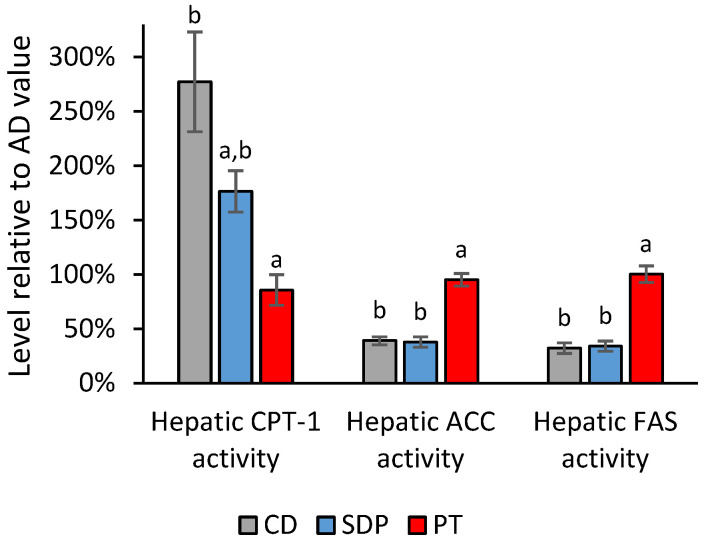
Effect of different treatments on hepatic activities of CPT-1, linked to fatty acid oxidation and obtaining energy, and ACC and FAS, linked to fatty acid synthesis, normalized to the values obtained in the AD group. Liver enzymatic activities of CPT-1, ACC, and FAS observed in the experimental groups of animals treated with AD supplemented with SDPs, or PT, and CD, normalized with respect to the levels obtained in the AD experimental group. Each bar represents the average ± SD of three experimental replicas that were carried out using *n* = 5 in each experimental group. In the figure, a: *p* < 0.05 versus the AD group and b: *p* < 0.05 versus the CD group.

Moreover, AD administration to the mice induced a decrease in the hepatic activity of the enzyme CPT-1, and an increase in the hepatic activity of the enzymes ACC and FAS, and the opposite effects were observed with CD administration. Consistent with the results in the transcripts, SDP supplementation to an AD significantly reversed the effect of an AD on these enzymes, and in the case of ACC and FAS, enzyme activity levels normalized to the levels observed with CD administration (Figure 9).

## 3. Discussion

**Figure 10:**
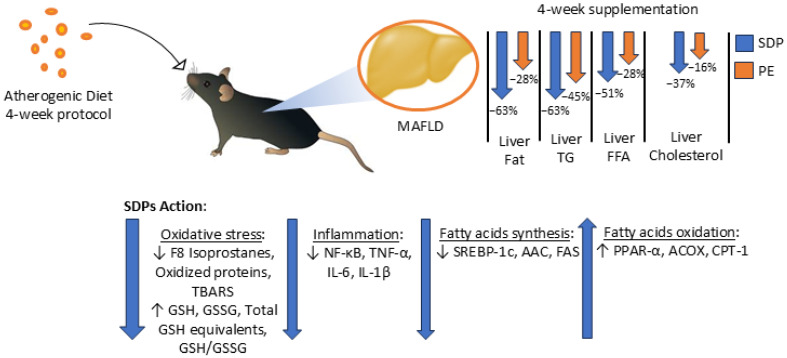
Summary of the results obtained in this study. Mice fed an AD during a 4-week protocol period developed MAFLD. Then, 4-week co-supplementation of AD with phytosterols in the form of SDPs, or PEs, was able to significantly improve Liver Fat, Liver TGs, Liver FFA, and Liver Cholesterol levels. The effectiveness of SDPs was observed to be significantly higher than that of PEs in regard to the aforementioned parameters. Additionally, to understand the action of SDPs, the metabolic parameters related to MAFLD were studied. It was observed that SDPs improved oxidative stress parameters (namely F8 Isoprostanes, TBARS, GSH, GSH equivalents, and the ratio of GSH/GSSG), inflammation parameters (namely NF-κB, TNF-α, IL-6, and IL-1β), fatty acid synthesis parameters (namely SREBP-1c, AAC, and FAS), and fatty acids oxidation parameters (PPAR-α, ACOX, and CPT-1). In the figure, arrows up and down indicate significant increase and decrease, respectively, of each set of parameters.

MAFLD is currently one of the most important causes of liver disease worldwide, with high liver-specific mortality rates [34]. Nevertheless, there is a poor awareness of the disease [35,36]. Furthermore, it is difficult to diagnose, since its symptoms appear only in an advanced stage of the disease. Nevertheless, no available pharmacology has been specifically approved for MAFLD treatment, and the available recommendations consist of lifestyle changes such as diet, weight loss, and physical activity [37]. Thus, recent research has been focused on natural products aimed at the amelioration of the mechanisms involved in MAFLD pathogenesis such as lipid synthesis, oxidation, and inflammation, as the only alternatives for treatment. In this study, a MAFLD model was induced in mice after AD feeding, and SDPs proved to be effective at reversing liver steatosis along with having an impact on several markers that might be involved in MAFLD pathogenesis, with more efficacy than conventional PEs. It is important to highlight that, here, a MAFLD reversion protocol was studied. Though some previous preclinical research focused on the prevention protocols of MAFLD with phytosterols [19,21,23,25], a couple of findings have demonstrated the reversion of liver inflammation [20,24]. More recently, Abo-Zaid et al. (2023) used free β-sitosterol to reverse liver steatosis [22]. Our current results reinforce the concept of using phytosterols for the treatment of MAFLD. These results are summarized in Figure 10.

### 3.1. Macro and Micro Effects of AD

As expected, mice fed an AD developed MAFLD and hepatic steatosis, as proven by histopathological analyses, due to the high levels of cholesterol and fat, and in particular saturated fat, in an AD compared to a CD. One of the major causes of hepatic steatosis is the increased absorption of fatty acids from plasma into the hepatocyte [4]. The source of increased fatty acids in plasma might be dietary or might also result from the disorders that are a part of the metabolic syndrome [38]. Once absorbed, FFA may be converted to TGs or oxidized as fuel. TGs can be then used for VLDL particle production and exported into plasma [4]. Interestingly, the AD-induced MAFLD produced no significant differences in mice body weight, liver weight, diet intake, and visceral adipose tissue. This has been previously described as a non-obese variant of the disease, and these results have been reported in humans [39] and in mice [40]. This was also observed by our group in a pilot scale study of human MAFLD patients [30].

### 3.2. Reversion by Phytosterols of Altered Liver Lipid Metabolism Parameters

SDP supplementation to an AD significantly reversed the AD-induced increases in liver fat, TGs, FFA, and cholesterol. This was further demonstrated by a liver histology analysis.

It is widely recognized that phytosterols are able to reduce cholesterol levels in the human body, and the main mechanisms behind this have been discussed elsewhere [41]. As for the TG-lowering effect of phytosterols, despite the fact that it is not well established, a meta-analysis proved that plant stanol esters were able to reduce serum TGs concentration [42], but only a few clinical trials have revealed the potential TG-lowering efficacy of free phytosterols in subjects with no hypertriglyceridemia [30,31,32]. The underlying mechanisms of phytosterols TG-lowering effect are unknown, but the following possibilities were hypothesized by Plat et al. (2009) [43]: (i) enhanced lipolysis mediated by lipoprotein lipase (LPL) which results in smaller amounts of TGs; (ii) increased cholesterol ester transfer protein (CETP) activity, which increases the transport of TGs from VLDL to HDL; and (iii) reduced hepatic TGs production and the concomitant production of TG-containing VLDL particles. In their study using PEs in humans, the authors discarded the possibility that the first two mechanisms were operating, since they noted that the activator and inhibitory ligands of LPL (Apo CII and apo CIII, respectively) were not affected by phytosterols, and neither the CETP mass nor the HDL-cholesterol concentration were affected. In the case of SDPs, in support of the first mechanism is the finding of a reduction in Apo B levels and the particle number of VLDL in the subjects with MAFLD treated for one year with SDPs, including the first evidence of reductions in liver steatosis being produced by phytosterols, which to date had not been previously demonstrated with PE supplementation in clinical studies [30]. Apo-B provides the scaffold for VLDL assembly [44], thus, a decrease in its concentration would generally result in a decreased secretion of VLDL particles. Moreover, a clinical study in metabolic syndrome patients comparing SDPs with PT showed consistent serum TGs reductions and VLDL-cholesterol reductions with a similar trend to that of TGs, and a decrease in waist circumference of up to 4 cm was observed in the SDP group, as opposed to no decrease as seen in the placebo group. Also, in accordance with Plat et al. [43], it was observed that the patients treated with SDPs tended to have lower quantities of VLDL particles [32]. Considering the second mechanism, in this study, although CETP was not assessed, HDL-cholesterol levels were affected by SDPs, but not by PEs, as observed by Plat et al. [43].

SDP supplementation to an AD produced a reversion in the liver oxidative stress parameters that were affected by AD-induced MAFLD. From the perspective of the multiple-hit theory, oxidative stress is a crucial step of the pathogenesis of MAFLD leading to inflammatory cascade activation [7]. This might be part of the therapeutic mechanism of SDPs in MAFLD patients.

### 3.3. Efficacy of SDPs Compared to PEs with Regard to Serum Metabolic Parameters

The AD-induced alterations of serum levels of TGs, as well as LDL-, HDL-, and total-cholesterol levels were significantly reversed by SDP supplementation to an AD. The conventional phytosterol formulation, PEs, produced significantly smaller reversions for the above-mentioned parameters, or no effect in the case of HDL-cholesterol levels. Accordingly, a clinical study in mild-hypercholesterolemic patients comparing SDPs with PEs, showed TGs and HDL-cholesterol level improvements with SDP treatment, as opposed to the unchanged values that occurred with PE treatment [31].

In regard to the results for the glucose and insulin levels, it is known that a diet high in fat affects glucose negatively [45]. Phytosterols supplementation to an AD, either in the form of SDPs or PEs, reversed an AD-induced increase in serum glucose levels. Consistently, SDP supplementation to an AD reversed significantly the AD-induced increases in serum insulin levels. Improvements in both parameters have been reported after phytosterol administration in diabetes-induced or hyperglycemic rats [46,47,48], as well as in humans [49,50]. A possible mechanism explaining the antidiabetic activity of phytosterols could be their interaction with membrane receptors and transporters such as the insulin receptor (1IRK), glucose transporter (GLUT4), and PPAR-γ, which was hypothesized after a demonstration in in silico simulations, and further supported by studies in rats demonstrating phytosterol’s similar anti-inflammatory and insulin-improving effectiveness to metformin. In this pathway, FXR could be a mediator [18].

The association of phytosterol supplementation with an anti-inflammatory effect is not clear in human studies [51]. Nevertheless, a significant reduction in CRP was observed in the patients with MAFLD after the consumption of SDPs for one year [30]. Thus, the anti-steatotic function of SDPs would be consistent with a detention of the inflammatory injury that stops the synthesis of liver acute phase proteins, such as CRP. Moreover, the already discussed results of the impacts on the liver oxidative stress parameters favor the hypothesis of the effect of phytosterols on liver inflammation.

Finally, in regard to the transaminase levels, MAFLD has been regarded as the most common cause of the asymptomatic elevation of transaminase levels [52]. GPT generally rises as an effect of liver injury, while GOT increases can also be caused by extrahepatic disorders. Though these parameters were not improved by SDP treatment in a pilot-scale clinical intervention [30], in the present study, the SDP supplementation to an AD significantly reversed the increase in the serum levels of the transaminases GPT and GOT, further reinforcing the importance of validating preclinical results with clinical studies.

### 3.4. Regulation of Metabolic and Inflammatory Responses by SDP

Considering the third mechanism of TGs reduction by phytosterols hypothesized by Plat et al. (2009) [43], in this study, SDP supplementation to an AD produced an increase in the transcript levels of the gene markers of lipid β-oxidation, PPAR-α, CPT-1, and ACOX,, and a decrease in transcript levels of the gene markers of fatty acid synthesis, SREBP-1c, ACC, and FAS. Moreover, the AD-induced increase in the levels of the liver markers of oxidation, such as liver TBARS, hepatic oxidized proteins, and liver F-8 Isoprostanes, was significantly reduced after SDP supplementation to an AD. As a possible upstream mechanism for these actions, it has been observed that stigmasterol antagonizes the Farnesoid X nuclear receptor (FXR), which mediates the activation of PPAR-α and the inhibition of SREBP-1c in the liver [18]. The above could be an additional pathway to the stigmasterol-mediated increase in bile acid synthesis detected in the mice MAFLD model [24].

The inhibition of enzymes and receptors that are crucial in the fatty acid oxidation and synthesis processes, might be part of the mechanisms through which fatty acids and TGs are increased in the liver and serum in MAFLD pathogenesis. The decreased mRNA transcript levels of PPAR-α, CTP-1, ACOX, and the increased mRNA transcript levels of ACC, FAS, and SREBP-1 have been previously reported in a mice model of MAFLD induced by a high fructose diet [53]. Consistently, SDP supplementation to an AD significantly reversed the decreased mRNA expression of the gene markers of lipid oxidation in liver tissue: PPAR-α, CTP-1 and ACOX. Consistently, SDP supplementation to an AD reversed an AD-induced decrease in hepatic CPT-1 enzymatic activity. PPAR-α is the master regulator of hepatic β-oxidation, while CPT-1 acts as a rate-limiting enzyme of the process in the mitochondria [54], and ACOX is the first and rate-limiting enzyme required for the peroxisomal β-oxidation process [55]. Fatty acid β-oxidation turns fatty acids in the liver into energy, thus reducing their concentration. Furthermore, SDP supplementation to an AD significantly reversed the increases in the hepatic transcript levels of the genetic markers of fatty acid synthesis: ACC, FAS, and SREBP-1c. Consistently, supplementing SDP to an AD reversed the induced increase in the hepatic levels of FAS and ACC enzymatic activities after AD feeding. SREBP-1c is a transcription factor located in the endoplasmic reticulum that regulates fatty acid synthesis [56]. SREBP-1c controls the expression of FAS [57], which is a crucial catalyzing enzyme in the last step of fatty acid synthesis, whereas ACC limits the rate at which acetyl-CoA is converted to malonyl-CoA, both of which are utilized by FAS for fatty acid synthesis [58].

In MAFLD, TGs accumulation in the hepatocytes can lead to cell damage through lipid peroxidation and the production of reactive oxygen species which lead to inflammation, among other effects [59,60]. Inflammation induction and the presence of the pro-inflammatory cytokines TNF-α, IL-6, and IL-1β, which are mediated by an increase in NF-κB, play an important part in the development of MAFLD [61]. An analysis of several studies has resulted in a positive correlation between IL-1β, IL-6, and TNF-α and MAFLD [62]. Moreover, the activity of the transcription factor NF-κB has been shown to be upregulated by the C-reactive protein (CRP) [63]. In relation to the anti-inflammatory role of SDP, its supplementation at least significantly counteracted the observed increase in the serum levels of TNF-α. Furthermore, the increased hepatic transcript levels of the genetic markers of inflammation, NF-κB, TNF-α, IL-6, and IL-1β, were also significantly reversed. The observed NF-κB reduction should be complementary to the NLRP3 decrease detected with stigmasterol for the downregulation of the active forms of cytokines [24]. Additionally, cytokines have been shown to be involved in further mechanisms of MAFLD pathogenesis [7]. For example, IL-1β might promote TGs and cholesterol accumulation and the development of lipid droplets in hepatocytes, thus promoting hepatic steatosis [64]. Although the IL-6 transcript level increase was reversed by SDP supplementation, a serum IL-6 decrease was not detected.

### 3.5. Strengths and Limitations of This Study

As also shown in this study, previous reports in rodent models of MAFLD have demonstrated the capacity of phytosterols, either free or esterified, to improve diverse plasma and liver lipids [19,20,21,22,24], serum inflammatory markers [19,21], and liver enzymes [21]. The transcript levels of the genes related to β-oxidation, fatty acid synthesis, and inflammation were also improved in previous MAFLD models in mice [19,20,21,22,24]. Nevertheless, no other formulation besides the SDP has been successful in the treatment of MAFLD patients [30]. Consistently, the present head-to-head preclinical comparison of the phytosterols formulated as SDPs or as PEs, not only showed the significantly higher biological activity of the former in the treatment of MAFLD, but also improved HDL-cholesterol levels, a serum parameter not shown to be affected before by the other formulations of phytosterols, nor by the PEs in this study. Paradoxically, phytosterol esters of α-linoleic acid were observed to present higher MAFLD prevention efficacy than crude free phytosterols. Han et al. (2019) showed that the plant sterol esters of α-linoleic acid show higher activity than crude free phytosterols [23], probably since the latter have a minimal affinity for the mixed micelle [65]. Differently to crude free phytosterols, formulating compounds as dispersions, as is the case with SDPs, is known to improve their water solubility [66], thus decreasing their precipitation in the intestinal lumen, and favoring their incorporation into the mixed micelle becoming more bioavailable than even phytosterol esters.

In addition to the health benefits of phytosterols, some drawbacks have also been described. Firstly, a recessive disorder known as sitosterolemia, where mutations in the ABCG5 and ABCG8 genes are probably involved, consists of an increased absorption and decreased excretion of phytosterols and cholesterol, and results in elevated serum concentrations of phytosterols, which might manifest as tendon and tuberous xanthomas and premature coronary atherosclerosis [67]. Nevertheless, it only affects individuals presenting these extremely rare mutations [68]. Secondly, some research has suggested that phytosterol consumption may affect the availability of the fat-soluble vitamins provided through a diet [69]; however, human interventions for short or long periods of time have consistently shown that this effect is not produced with SDP administration [30,31,32]. Specifially for vitamin D availability, as discussed in Brañes et al. [30], any condition improving liver function, such as SDP supplementation, might improve metabolic performance, which includes the transformation of cholecalciferol into 25-hydroxy-cholecalciferol which is determined in plasma [70].

Some limitations in this study include the fact that results in mice do not necessarily traduce into results in humans, although they do indicate a possibility of activity. Therefore, these results must be contrasted with the clinical studies studying the interventions for MAFLD patients with SDPs and PE. Additionally, much of the metabolic parameters in this study were only studied in the SDP treatment group, but not in the PE treatment group. Further studies comparing metabolic parameters (i.e., oxidation, fatty acid synthesis, and inflammation parameters) and behaviors between both treatments would be useful to understand the mechanisms behind the increased activity of SDPs compared to PEs that was observed in this study.

## 4. Materials and Methods

### 4.1. Materials

Water-soluble SDPs (Cardiosmile™, Inversiones Nutrartis Ltd., Santiago, Chile) and PEs (Vitasterol™ S-80 esterified-SF non-GMO, Vitae Naturals, Toledo, Spain) were used as sources of phytosterols. SDPs contain free phytosterols of pine tree origins with the following composition: 70–80% β-sitosterol; <15% β-sitostanol; <15% campesterol; <5% campestanol; and <2% stigmasterol. PEs contain pine tree and rapeseed esterified phytosterols with the following composition: <80% β-sitosterol; <40% campesterol; <30% stigmasterol; <15% β-sitostanol; <5% campestanol; <3% brassicasterol; and <3% other sterols/stanols. PT consisted of a mixture of the excipients of Cardiosmile plus additional stabilizing components (titanium dioxide, xanthan gum, carrageenan, surfactants (<0.5%), potassium sorbate, citric acid, and water) without the active compound (phytosterols).

### 4.2. Animal Model and Treatment

MAFLD was induced following the protocol provided by Rowles III et al. (2018) [71]. Briefly, male mice weighing 20–21 g (C57BL/6J from Bioterio Central, ICBM, Faculty of Medicine, University of Chile) were fed for 4 weeks with a high fat and cholesterol diet (Research Diets Inc., New Brunswick, NJ, USA), herein identified as an atherogenic diet (AD) composed of 20.8% protein, 15.5% fat, 6.1% carbohydrates, and 1.25% cholesterol. Mice in the control group were fed for 8 weeks the Mouse Diet #5015 (LabDiet, Inc., Los Angeles, CA, USA), herein identified as control diet (CD), which was composed of 18.9% protein, 11.1% fat, 51.8% carbohydrates, and 0.003% cholesterol.

**Figure 11:**
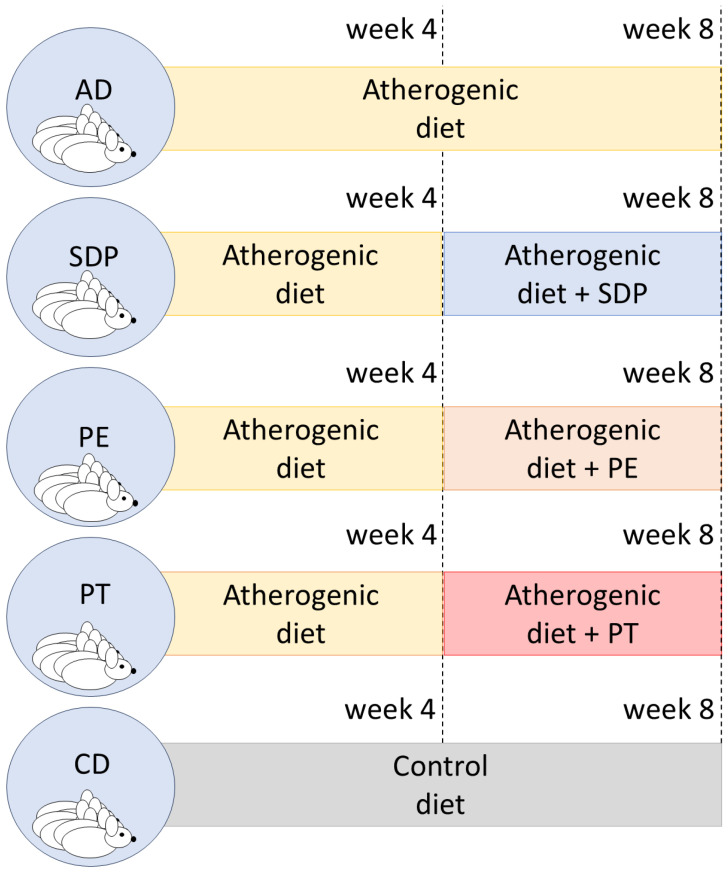
Experimental design used in this study. Five experimental groups were considered following the protocol of fatty liver generation with atherogenic diet (AD) for four weeks, and then another four weeks with AD alone or supplementation with a submicron dispersion of phytosterols (SDPs), or phytosterol esters (PEs) or placebo (PT). Another group was fed for eight weeks with control diet.

Prior to mixing AD with SDP, PE, or PT, diet pellets were ground and homogenized with a minipimer (Braun, MR 404 Plus 300 W, Kronberg im Taunus, Germany). Then, SDPs, melted PEs, or PT, were added to the diets along with a sufficient amount of water to form a homogeneous mass, moldable into cylindrical tubes. SDPs or PEs were mixed an AD for a final 2% weight concentration of free phytosterols. This dosage was selected considering the protocols of previous studies using phytosterols in mice to ensure safety and effectiveness [20,23,24] and PT was mixed an AD using a total weight equivalent to that used for SDPs, replicating the amount of product and excipients in active treatments. Finally, the cylinders were dried under vacuum at 60 °C until a constant weight was obtained. These were provided as supplemented food during the following 4-week additional period, while in parallel, a negative-control group labelled “AD”, continued with AD feeding for the 4-week additional period (Figure 11).

At the end of the 8th week, animals were fasted with free access to water for 5 h and anesthetized with isoflurane (Lunan Baxter Pharmaceuticals Co. Ltd., Shandong, China) prior to euthanasia. Blood was extracted through cardiac puncture for serum biochemical parameter assessments. Liver samples were either frozen in liquid nitrogen until use for biochemical determinations and mRNA extraction, or fixed in phosphate-buffered formalin, embedded in paraffin for a histological analysis of steatosis, inflammation, and fibrosis after staining with hematoxylin-eosin (HE). Results provided correspond to the average ± SD of three independent experiments that were carried out using n = 5 in each experimental group. Liver steatosis scores were then calculated according to Brunt et al., 1999 [33].

#### 4.2.1. Ethics

All animal procedures described in this study are in strict compliance with the Guide for the Care and Use of Laboratory Animals (National Academy of Sciences, NIH Publication 6–23, revised 1985) and were approved by the Bioethics Committee for Research in Animals, Faculty of Medicine, University of Chile (CBA#1240 MED UCH).

#### 4.2.2. Biochemical Parameters

The biochemical parameter assessment was conducted as previously described [72]. Total fat content (%) in liver was evaluated according to the Bligh and Dyer method [73]. Briefly, liver samples were homogenized with ice-cold PBS using an UltraTurrax homogenizer (Janke & Kunkel, Stufen, Germany) and then extracted twice with chloroform/methanol (2:1 *v*/*v*) containing 0.01% butylated hydroxytoluene (BHT). Total lipids were recovered from the chloroform phase. FFA (μmol/g liver) and TGs (mg/g liver) levels were measured using specific kits, according to the manufacturer’s instructions (Cayman Chemical Company, Ann Arbor, MI, USA). Cholesterol content in the liver (mg/g liver) was measured using a specific kit according to the manufacturer’s instructions (Abcam Inc, Toronto, ON, Canada). GSH (μmol/g liver) and GSSG (μmol/g liver) contents were assessed with an enzymatic recycling method [74]. Liver TBARS (nmol/mg protein), hepatic oxidized proteins (nmol/mg protein) and hepatic F-8 isoprostanes (pg/mg liver) were determined by colorimetric assays according to the manufacturer’s instructions (Cayman Chemical Company, Ann Arbor, MI, USA). Serum TGs (mg/dL), total-cholesterol (mg/dL), LDL-cholesterol (mg/dL), HDL-cholesterol (mg/dL), and glucose (mg/dL) levels were measured using specific kits according to the manufacturer’s instructions (Wiener Lab, Rosario, Argentina). Serum insulin levels (units/mL) were measured with a commercial immunoassay kit for mice according to the manufacturer’s instructions (Mercodia, Uppsala, Sweden). Serum GOT and GPT activities (U/L) were measured using specific diagnostic kits according to the manufacturer’s instructions (Biomerieux SA, Marcy l’Etoile, France). ELISA kits were used for the assessment of serum levels (pg/mL) of tumor necrosis factor-α (TNF-α) and interleukin-6 (IL-6) according to the manufacturer’s instructions (Cayman Chemical Company, Ann Arbor, MI, USA).

#### 4.2.3. Real-Time Quantitative PCR

The quantification of transcripts was conducted as previously described [75]. From the liver samples, total RNA was extracted using Trizol (Invitrogen, Paisley, UK). A total of 2 µg of RNA were obtained, and then treated with DNase (DNA free kit; Ambion, Austin, TX, USA). First strand cDNAs were generated from treated RNA with M-MLV reverse transcriptase (Invitrogen, Paisley, UK), utilizing random hexamers (Invitrogen, Paisley, UK) and a dNTP mix (Bioline, London, UK). The obtained cDNA was amplified with specific primers for rats in a total volume of 10 µL. Primers were optimized to yield 95–100% of reaction efficiency with PCR products run on agarose gel electrophoresis to verify the correct amplification length. The formation of a single desired PCR product in each PCR reaction was verified through melt curve analyses. The expression level of each sample was normalized against the expression level of glyceraldehyde-3-phosphate dehydrogenase (GAPDH) as the internal control. The relative expression level was calculated using the comparative CT method 2(-ΔΔCT) and values were normalized to the GAPDH level. Real-time quantitative PCR (qPCR) was performed in a Stratagen Mx3000P system (Agilent Technologies, Santa Clara, CA, USA) using Brilliant II SYBR Green QPCR Master Mix (Agilent Technologies, Santa Clara, CA, USA).

#### 4.2.4. Determination of Enzyme Activities in Liver

The activities of ACC (pkat/mg g protein) [76], FAS (pkat/mg g protein) [77], and CPT-1 (pkat/mg g protein) [78] were determined spectrophotometrically.

#### 4.2.5. Statistical Analyses

Parameter values were averaged for each experimental group, and standard deviations were calculated. These results were subjected to ANOVA with Bonferroni correction to explore if statistically significant differences were produced between groups. For these analyses, an α value of 0.05 was selected.

## 5. Conclusions

This study supports phytosterol’s therapeutic potential for the treatment of MAFLD in mice, and their reversion of alterations in the specific metabolic parameters that are part of this pathology, related to lipid metabolism, glucose metabolism, and inflammation. Moreover, this study underlines the superiority of phytosterols formulated as SDPs compared to PEs in the reversion of alterations to the parameters that are characteristic of MAFLD. It must be stressed out that further studies focused on humans are needed to confirm the findings of this study. In particular, clinical studies comparing the effect of SDPs and PEs on MAFLD treatment would help to support our hypotheses.

## Figures and Tables

**Table 1:** Tabulation of body, liver, and visceral tissue weight and diet intake. Atherogenic diet (AD)-induced mice metabolic dysfunction-associated fatty liver disease (MAFLD) model alone, co-supplemented with submicron dispersions of phytosterols (SDPs) or with placebo (PT), and compared with control diet (CD). Results correspond to the average ± SD of three independent experiments that were carried out using *n* = 5 in each experimental group.

Study Group	Initial Weight (g)	Final Weight (g)	Initial Diet Intake (g)	Final Diet Intake (g)	Liver Weight (g)	Visceral Adipose Tissue Weight (g)
AD	20.5 ± 1.3	24.5 ± 1.9	3.4 ± 0.8	3.7 ± 0.9	1.6 ± 0.1	0.7 ± 0.4
CD	20.8 ± 1.5	23.8 ± 2.2	3.6 ± 0.9	3.4 ± 0.7	1.6 ± 0.2	0.7 ± 0.2
SDP	19.8 ± 1.6	23.8 ± 2.6	3.1 ± 0.6	3.3 ± 1.1	1.7 ± 0.2	0.7 ± 0.2
PT	20.7 ± 1.8	25.5 ± 2.7	3.5 ± 1.8	3.3 ± 0.9	1.6 ± 0.1	0.7 ± 0.3

## Data Availability

Data contained within the article.

## References

[1] Arguello G., Balboa E., Arrese M., Zanlungo S. (2015). Recent insights on the role of cholesterol in non-alcoholic fatty liver disease. Biochim. Biophys. Acta. Mol. Basis Dis..

[2] Sangro P., de la Torre Aláez M., Sangro B., D’Avola D. (2023). Metabolic dysfunction–associated fatty liver disease (MAFLD): An update of the recent advances in pharmacological treatment. J. Physiol. Biochem..

[3] Flisiak-Jackiewicz M., Bobrus-Chociej A., Wasilewska N., Lebensztejn D.M. (2021). From Nonalcoholic Fatty Liver Disease (NAFLD) to Metabolic Dysfunction-Associated Fatty Liver Disease (MAFLD)—New Terminology in Pediatric Patients as a Step in Good Scientific Direction?. J. Clin. Med..

[4] Bradbury M.W. (2006). Lipid metabolism and liver inflammation. I. Hepatic fatty acid uptake: Possible role in steatosis. Am. J. Physiol. Gastrointest. Liver Physiol..

[5] Drescher H.K., Weiskirchen S., Weiskirchen R. (2019). Current status in testing for nonalcoholic fatty liver disease (NAFLD) and nonalcoholic steatohepatitis (NASH). Cells.

[6] Maurice J., Manousou P. (2018). Non-alcoholic fatty liver disease. Clin. Med..

[7] Buzzetti E., Pinzani M., Tsochatzis E.A. (2016). The multiple-hit pathogenesis of non-alcoholic fatty liver disease (NAFLD). Metab. Clin. Exp..

[8] Guilherme A., Virbasius J.V., Puri V., Czech M.P. (2008). Adipocyte dysfunctions linking obesity to insulin resistance and type 2 diabetes. Nat. Rev. Mol. Cell Biol..

[9] Branković M., Jovanović I., Dukić M., Radonjić T., Oprić S., Klašnja S., Zdravković M. (2022). Lipotoxicity as the leading cause of non-alcoholic steatohepatitis. Int. J. Mol. Sci..

[10] Kirpich I.A., Marsano L.S., McClain C.J. (2015). Gut–liver axis, nutrition, and non-alcoholic fatty liver disease. Clin. Biochem..

[11] Tarantino G., Citro V., Capone D. (2019). Nonalcoholic fatty liver disease: A challenge from mechanisms to therapy. J. Clin. Med..

[12] Zeng X.F., Varady K.A., Wang X.D., Targher G., Byrne C.D., Tayyem R., Latella G., Bergheim I., Valenzuela R., George J. (2024). The role of dietary modification in the prevention and management of metabolic dysfunction-associated fatty liver disease: An international multidisciplinary expert consensus. Metabolism.

[13] Fujii H., Kawada N., Japan Study Group of Nafld (JSG-NAFLD) (2020). The role of insulin resistance and diabetes in nonalcoholic fatty liver disease. Int. J. Mol. Sci..

[14] Ratziu V., Bellentani S., Cortez-Pinto H., Day C., Marchesini G. (2010). A position statement on NAFLD/NASH based on the EASL 2009 special conference. J. Hepatol..

[15] Zhao M., Chen S., Ji X., Shen X., You J., Liang X., Yin H., Zhao L. (2021). Current innovations in nutraceuticals and functional foods for intervention of non-alcoholic fatty liver disease. Pharmacol. Res..

[16] Guirguis E., Dougherty J., Thornby K., Grace Y., Mack K. (2024). Resmetirom: The First Food and Drug Administration–Approved Medication for Nonalcoholic Steatohepatitis (NASH). Ann. Pharmacother..

[17] Frasinariu O., Serban R., Trandafir L.M., Miron I., Starcea M., Vasiliu I., Alisi A., Temneanu O.R. (2022). The role of phytosterols in nonalcoholic fatty liver disease. Nutrients.

[18] Brañes M.C., Gillet R., Valenzuela R. (2024). Nuclear receptors behind the therapeutic effects of plant sterols on metabolism: A review. Lipids.

[19] Song L., Qu D., Zhang Q., Jiang J., Zhou H., Jiang R., Li Y., Zhang Y., Yan H. (2017). Phytosterol esters attenuate hepatic steatosis in rats with non-alcoholic fatty liver disease rats fed a high-fat diet. Sci. Rep..

[20] Plat J., Hendrikx T., Bieghs V., Jeurissen M.L., Walenbergh S.M., van Gorp P.J., De Smet E., Konings M., Vreugdenhil A.C., Guichot Y.D. (2014). Protective role of plant sterol and stanol esters in liver inflammation: Insights from mice and humans. PLoS ONE.

[21] Han H., Ma H., Rong S., Chen L., Shan Z., Xu J., Zhang Y., Liu L. (2015). Flaxseed oil containing flaxseed oil ester of plant sterol attenuates high-fat diet-induced hepatic steatosis in apolipoprotein-E knockout mice. J. Funct. Foods.

[22] Abo-Zaid O.A., Moawed F.S., Ismail E.S., Farrag M.A. (2023). β-sitosterol attenuates high-fat diet-induced hepatic steatosis in rats by modulating lipid metabolism, inflammation and ER stress pathway. BMC Pharmacol. Toxicol..

[23] Han H., Guo Y., Li X., Shi D., Xue T., Wang L., Li Y., Zheng M. (2019). Plant Sterol Ester of α-Linolenic Acid Attenuates Nonalcoholic Fatty Liver Disease by Rescuing the Adaption to Endoplasmic Reticulum Stress and Enhancing Mitochondrial Biogenesis. Oxid. Med. Cell Longev..

[24] Xin Y., Li X., Zhu X., Lin X., Luo M., Xiao Y., Ruan Y., Guo H. (2023). Stigmasterol protects against steatohepatitis induced by high-fat and high-cholesterol diet in mice by enhancing the alternative bile acid synthesis pathway. J. Nutr..

[25] Song L., Qu D., Ouyang P., Ding X., Wu P., Guan Q., Yang L. (2020). The regulatory effects of phytosterol esters (PSEs) on gut flora and faecal metabolites in rats with NAFLD. Food Funct..

[26] Mohammad Shahi M., Javanmardi M.A., Seyedian S.S., Haghighizadeh M.H. (2018). Effects of phytosterol supplementation on serum levels of lipid profiles, liver enzymes, inflammatory markers, adiponectin, and leptin in patients affected by nonalcoholic fatty liver disease: A double-blind, placebo-controlled, randomized clinical trial. J. Am. Coll. Nutr..

[27] Cicero A.F., Fogacci F., Bove M., Veronesi M., Rizzo M., Giovannini M., Borghi C. (2017). Short-term effects of a combined nutraceutical on lipid level, fatty liver biomarkers, hemodynamic parameters, and estimated cardiovascular disease risk: A double-blind, placebo-controlled randomized clinical trial. Adv. Ther..

[28] Song L., Zhao X.G., Ouyang P.L., Guan Q., Yang L., Peng F., Du H., Yin F., Yan W., Yu W.J. (2020). Combined effect of n-3 fatty acids and phytosterol esters on alleviating hepatic steatosis in non-alcoholic fatty liver disease subjects: A double-blind placebo-controlled clinical trial. Br. J. Nutr..

[29] Chen D.-L., Huang P.-H., Chiang C.-H., Leu H.-B., Chen J.-W., Lin S.-J. (2015). Phytosterols increase circulating endothelial progenitor cells and insulin-like growth factor-1 levels in patients with nonalcoholic fatty liver disease: A randomized crossover study. J. Funct. Foods.

[30] Brañes M.C., Gillet R., Valenzuela R. (2023). Efficacy of Submicron Dispersible Free Phytosterols on Non-Alcoholic Fatty Liver Disease: A Pilot Study. J. Clin. Med..

[31] Shaghaghi M.A., Harding S.V., Jones P.J. (2014). Water dispersible plant sterol formulation shows improved effect on lipid profile compared to plant sterol esters. J. Funct. Foods.

[32] Palmeiro-Silva Y.K., Aravena R.I., Ossio L., Parro Fluxa J. (2020). Effects of daily consumption of an aqueous dispersion of free-phytosterols nanoparticles on individuals with metabolic syndrome: A randomised, double-blind, placebo-controlled clinical trial. Nutrients.

[33] Brunt E.M., Janney C.G., Di Bisceglie A.M., Neuschwander-Tetri B.A., Bacon B.R. (1999). Nonalcoholic steatohepatitis: A proposal for grading and staging the histological lesions. Am. J. Gastroenterorol..

[34] Younossi Z.M., Koenig A.B., Abdelatif D., Fazel Y., Henry L., Wymer M. (2016). Global epidemiology of nonalcoholic fatty liver disease—Meta-analytic assessment of prevalence, incidence, and outcomes. Hepatology.

[35] Alqahtani S.A., Paik J.M., Biswas R., Arshad T., Henry L., Younossi Z.M. (2021). Poor awareness of liver disease among adults with NAFLD in the United States. Hepatol. Commun..

[36] Ghevariya V., Sandar N., Patel K., Ghevariya N., Shah R., Aron J., Anand S. (2014). Knowing what’s out there: Awareness of non-alcoholic fatty liver disease. Front. Med..

[37] Lazarus J.V., Mark H.E., Anstee Q.M., Arab J.P., Batterham R.L., Castera L., Cortez-Pinto H., Crespo J., Cusi K., Dirac M.A. (2022). Advancing the global public health agenda for NAFLD: A consensus statement. Nat. Rev. Gastroenterol. Hepatol..

[38] Donnelly K.L., Smith C.I., Schwarzenberg S.J., Jessurun J., Boldt M.D., Parks E.J. (2005). Sources of fatty acids stored in liver and secreted via lipoproteins in patients with nonalcoholic fatty liver disease. J. Clin. Investig..

[39] Yasutake K., Nakamuta M., Shima Y., Ohyama A., Masuda K., Haruta N., Fujino T., Aoyagi Y., Fukuizumi K., Yoshimoto T. (2009). Nutritional investigation of non-obese patients with non-alcoholic fatty liver disease: The significance of dietary cholesterol. Scand. J. Gastroenterol..

[40] Tu L.N., Showalter M.R., Cajka T., Fan S., Pillai V.V., Fiehn O., Selvaraj V. (2017). Metabolomic characteristics of cholesterol-induced non-obese nonalcoholic fatty liver disease in mice. Sci. Rep..

[41] Jiménez P., Bustamante A., Echeverría F., Sambra V., Rincón-Cervera M.Á., Farías C., Valenzuela R. (2024). Metabolic Benefits of Phytosterols: Chemical, Nutritional, and Functional Aspects. Food Rev. Int..

[42] Naumann E., Plat J., Kester A.D., Mensink R.P. (2008). The baseline serum lipoprotein profile is related to plant stanol induced changes in serum lipoprotein cholesterol and triacylglycerol concentrations. J. Am. Coll. Nutr..

[43] Plat J., Brufau G., Dallinga-Thie G.M., Dasselaar M., Mensink R.P. (2009). A plant stanol yogurt drink alone or combined with a low-dose statin lowers serum triacylglycerol and non-HDL cholesterol in metabolic syndrome patients. J. Am. Coll. Nutr..

[44] Sniderman A.D., Thanassoulis G., Glavinovic T., Navar A.M., Pencina M., Catapano A., Ference B.A. (2019). Apolipoprotein B particles and cardiovascular disease: A narrative review. JAMA Cardiol..

[45] Lichtenstein A.H., Schwab U.S. (2000). Relationship of dietary fat to glucose metabolism. Atherosclerosis.

[46] Gupta R., Sharma A.K., Dobhal M., Sharma M., Gupta R. (2011). Antidiabetic and antioxidant potential of β-sitosterol in streptozotocin-induced experimental hyperglycemia. J. Diabetes..

[47] Ramalingam S., Packirisamy M., Karuppiah M., Vasu G., Gopalakrishnan R., Gothandam K., Thiruppathi M. (2020). Effect of β-sitosterol on glucose homeostasis by sensitization of insulin resistance via enhanced protein expression of PPRγ and glucose transporter 4 in high fat diet and streptozotocin-induced diabetic rats. Cytotechnology.

[48] Ivorra M., D’ocon M., Paya M., Villar A. (1988). Antihyperglycemic and insulin-releasing effects of beta-sitosterol 3-beta-D-glucoside and its aglycone, beta-sitosterol. Arch. Int. Pharmacodyn. Ther..

[49] Salehi-Sahlabadi A., Varkaneh H.K., Shahdadian F., Ghaedi E., Nouri M., Singh A., Farhadnejad H., Găman M.-A., Hekmatdoost A., Mirmiran P. (2020). Effects of Phytosterols supplementation on blood glucose, glycosylated hemoglobin (HbA1c) and insulin levels in humans: A systematic review and meta-analysis of randomized controlled trials. J. Diabetes. Metab. Disord..

[50] Li Q., Xing B. (2016). A phytosterol-enriched spread improves lipid profile and insulin resistance of women with gestational diabetes mellitus: A randomized, placebo-controlled double-blind clinical trial. Diabetes Technol. Ther..

[51] Rocha V.Z., Ras R.T., Gagliardi A.C., Mangili L.C., Trautwein E.A., Santos R.D. (2016). Effects of phytosterols on markers of inflammation: A systematic review and meta-analysis. Atherosclerosis.

[52] Oh R.C., Hustead T.R., Ali S.M., Pantsari M.W. (2017). Mildly elevated liver transaminase levels: Causes and evaluation. Am. Fam. Physician.

[53] Shi L.-j., Shi L., Song G.-y., Zhang H.-f., Hu Z.-j., Wang C., Zhang D.-h. (2013). Oxymatrine attenuates hepatic steatosis in non-alcoholic fatty liver disease rats fed with high fructose diet through inhibition of sterol regulatory element binding transcription factor 1 (Srebf1) and activation of peroxisome proliferator activated receptor alpha (Pparα). Eur. J. Pharmacol..

[54] Zammit V.A. (2008). Carnitine palmitoyltransferase 1: Central to cell function. IUBMB Life.

[55] Reddy J.K., Hashimoto T. (2001). Peroxisomal β-oxidation and peroxisome proliferator–activated receptor α: An adaptive metabolic system. Annu. Rev. Nutr..

[56] Colgan S.M., Al-Hashimi A.A., Austin R.C. (2011). Endoplasmic reticulum stress and lipid dysregulation. Expert. Rev. Mol. Med..

[57] Yang Y., Li W., Liu Y., Sun Y., Li Y., Yao Q., Li J., Zhang Q., Gao Y., Gao L. (2014). Alpha-lipoic acid improves high-fat diet-induced hepatic steatosis by modulating the transcription factors SREBP-1, FoxO1 and Nrf2 via the SIRT1/LKB1/AMPK pathway. J. Nutr. Biochem..

[58] Chirala S.S., Wakil S.J. (2004). Structure and function of animal fatty acid synthase. Lipids.

[59] Ma Y., Lee G., Heo S.Y., Roh Y.S. (2021). Oxidative stress is a key modulator in the development of nonalcoholic fatty liver disease. Antioxidants.

[60] Tilg H., Moschen A.R. (2010). Evolution of inflammation in nonalcoholic fatty liver disease: The multiple parallel hits hypothesis. Hepatology.

[61] Wandji L.C.N., Gnemmi V., Mathurin P., Louvet A. (2020). Combined alcoholic and non-alcoholic steatohepatitis. JHEP Rep..

[62] Duan Y., Pan X., Luo J., Xiao X., Li J., Bestman P.L., Luo M. (2022). Association of inflammatory cytokines with non-alcoholic fatty liver disease. Front. Immunol..

[63] Bian F., Yang X.-Y., Xu G., Zheng T., Jin S. (2019). CRP-induced NLRP3 inflammasome activation increases LDL transcytosis across endothelial cells. Front. Pharmacol..

[64] Mirea A.-M., Tack C.J., Chavakis T., Joosten L.A., Toonen E.J. (2018). IL-1 family cytokine pathways underlying NAFLD: Towards new treatment strategies. Trends Mol. Med..

[65] Feng S., Wang L., Shao P., Sun P., Yang C.S. (2022). A review on chemical and physical modifications of phytosterols and their influence on bioavailability and safety. Crit. Rev. Food. Sci. Nutr..

[66] Vasconcelos T., Sarmento B., Costa P. (2007). Solid dispersions as strategy to improve oral bioavailability of poor water soluble drugs. Drug Discov. Today.

[67] Salen G., Patel S., Batta A.K. (2002). Sitosterolemia. Cardiovasc. Drug Rev..

[68] Tada H., Nomura A., Ogura M., Ikewaki K., Ishigaki Y., Inagaki K., Tsukamoto K., Dobashi K., Nakamura K., Hori M. (2021). Diagnosis and management of sitosterolemia 2021. J. Atheroscler. Thromb..

[69] Baumgartner S., Ras R.T., Trautwein E.A., Mensink R.P., Plat J. (2017). Plasma fat-soluble vitamin and carotenoid concentrations after plant sterol and plant stanol consumption: A meta-analysis of randomized controlled trials. Eur. J. Nutr..

[70] Kanis J. (1982). Vitamin D metabolism and its clinical application. J. Bone Jt. Surg. Br. Vol..

[71] Rowles III J.L., Han A., Miller R.J., Kelly J.R., Applegate C.C., Wallig M.A., O’Brien W.D., Erdman J.W. (2019). Low fat but not soy protein isolate was an effective intervention to reduce nonalcoholic fatty liver disease progression in C57BL/6J mice: Monitored by a novel quantitative ultrasound (QUS) method. Nutr. Res..

[72] Valenzuela R., Espinosa A., Llanos P., Hernandez-Rodas M.C., Barrera C., Vergara D., Romero N., Pérez F., Ruz M., Videla L.A. (2016). Anti-steatotic effects of an n-3 LCPUFA and extra virgin olive oil mixture in the liver of mice subjected to high-fat diet. Food Funct..

[73] Bligh E.G., Dyer W.J. (1959). A rapid method of total lipid extraction and purification. Can. J. Biochem. Physiol..

[74] Rahman I., Kode A., Biswas S.K. (2006). Assay for quantitative determination of glutathione and glutathione disulfide levels using enzymatic recycling method. Nat. Protoc..

[75] Hernández-Rodas M.C., Valenzuela R., Echeverría F., Rincón-Cervera M.Á., Espinosa A., Illesca P., Muñoz P., Corbari A., Romero N., Gonzalez-Mañan D. (2017). Supplementation with docosahexaenoic acid and extra virgin olive oil prevents liver steatosis induced by a high-fat diet in mice through PPAR-α and Nrf2 upregulation with concomitant SREBP-1c and NF-kB downregulation. Mol. Nutr. Food Res..

[76] Zimmermann R., Haemmerle G., Wagner E.M., Strauss J.G., Kratky D., Zechner R. (2003). Decreased fatty acid esterification compensates for the reduced lipolytic activity in hormone-sensitive lipase-deficient white adipose tissue. J. Lipid Res..

[77] Halestrap A.P., Denton R.M. (1973). Insulin and the regulation of adipose-tissue acetyl-coenzyme A carboxylase. Biochem. J..

[78] Karlic H., Lohninger S., Koeck T., Lohninger A. (2002). Dietary l-carnitine stimulates carnitine acyltransferases in the liver of aged rats. J. Histochem. Cytochem..

